# AKAP6 controls NFATc4 activity for BDNF-mediated neuroprotection

**DOI:** 10.1186/s13041-024-01157-8

**Published:** 2024-11-22

**Authors:** Joanna Mackiewicz, Julia Tomczak, Malwina Lisek, Feng Guo, Tomasz Boczek

**Affiliations:** 1 Department of Molecular Neurochemistry, Medical University of Lodz, Lodz, Poland; 2 Department of Pharmaceutical Toxicology, China Medical University, Shenyang, China; 3 Department of Pharmacy, The Fourth Affiliated Hospital of China Medical University, Shenyang, China

**Keywords:** BDNF, AKAP6, NFATc4, Calcineurin, Neuroprotection

## Abstract

Brain-derived neurotrophic factor (BDNF) is known for its potent prosurvival effect. Despite successfully replicating this effect in various clinical and pre-clinical models, the complete characterization of the molecular mechanisms underlying its neuroprotective action remains incomplete. Emerging research suggests a vital role for A-kinase anchoring proteins (AKAPs) as central nodal points orchestrating BDNF-dependent signaling. Among the over 50 identified AKAPs, AKAP6 has recently gained special attention due to its involvement in the neurotrophin-mediated survival of injured retinal ganglion cells (RGCs). However, the mechanisms by which AKAP6 responds to pro-survival BDNF signaling remain unknown. In this study, we shown that AKAP6 plays a crucial role in regulating BDNF-mediated NFAT transcriptional activity in neuronal survival by anchoring protein phosphatase calcineurin (CaN) and nuclear factor of activated T cells (NFATc4). Furthermore, we demonstrate that disrupting the anchoring of CaN diminishes the pro-survival effect of BDNF. Lastly, through experiments with NFATc4−/− mice, we provide evidence that NFATc4 acts downstream to BDNF’s neuroprotection in vivo. These findings could offer valuable insights for developing neuroprotective strategies aimed at preserving injured neurons from degeneration and promoting their regeneration.

**Supplementary Information:**

The online version contains supplementary material available at 10.1186/s13041-024-01157-8.

## Introduction

It is now becoming apparent that neuronal degeneration is, at least in part, attributable to insufficient trophic signaling, as the deprivation of essential neurotrophins leads to the induction of apoptosis [1]. One of the most powerful trophic factors is BDNF. The exact mechanism of BDNF-mediated neuroprotection remains unclear but it likely involves a crosstalk between Ca^2+^-dependent signaling and mitogen activated protein kinase (MAPK) cascades [1]. Growing body of evidence suggests that BDNF may counterbalance the pro-apoptotic signaling and/or affect the expression of specific set of genes supporting neuronal survival or delaying axonal degeneration [2]. This complexity of action requires a precise, spatially and timely-resolved orchestration of different, frequently opposite signals by highly specialized multimeric signalosomes, providing a control over transcriptional mechanisms and acting as a switch between survival and apoptosis.

AKAP6 (mAKAP, 250 kDa alternatively-spliced α-isoform) is a scaffold protein located in the perinuclear space in hippocampal neurons and RGCs [3, 4], that has been originally identified as a scaffold for protein kinase A (PKA). Besides PKA, it binds a large number of enzymes involved in stress responses including CaN and CaN-dependent transcription factor–NFAT [5]. Unlike the function of muscle-specific shorter β isoform, the function of neuronal AKAP6 has not been widely studied. A recent report recognized AKAP6 as a scaffold mediating signaling in stressed neurons that is required for in vivo neuroprotection [3]. In AKAP6-deficient mice, BDNF did not promote RGC survival after optic nerve crush (ONC), suggesting AKAP6 involvement in BDNF-mediated pro-survival signaling [3]. However, how AKAP6 coordinates signaling pathways involved in BDNF-mediated neuroprotection remains unresolved.

## Materials and methods

The detailed descriptions of materials and methods used in this study can be found in Supplementary file 1.

## Results

**Fig. 1:**
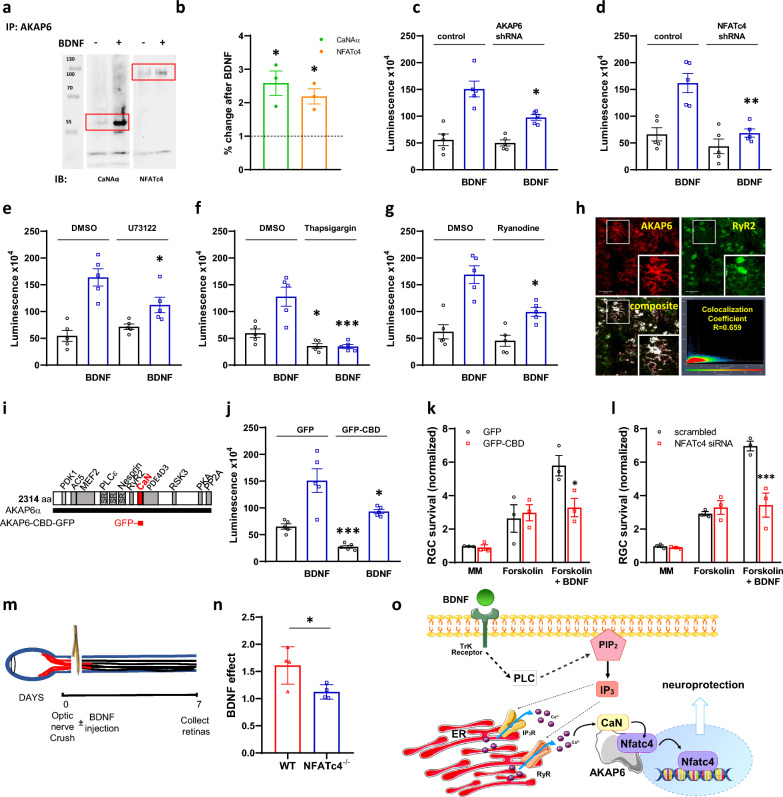
AKAP6 regulates NFAT activity for BDNF-mediated neuronal survival. **A** The AKAP6 antibody was used for immunoprecipitation of endogenous calcineurin (catalytic Aα subunit, CaN) or NFATc4 proteins from primary hippocampal neurons following incubation with BDNF. All presented blots are representative of experiments conducted at least three times. **B** Quantitative densitometric analysis of band intensity. The results are expressed in arbitrary units, defined as the optical density per milligram of protein relative to vehicle-treated cells, which were set as 100%, n = 3. **C** Primary hippocampal neurons were co-transduced with NFAT dual-reporter lentivirus and Lenti-AKAP6 shRNA (or an appropriate control), or alternatively, with **D** Lenti-NFATc4 shRNA (or an appropriate control). NFAT transcriptional activity was assessed in cell lysates following BDNF stimulation by quantifying luciferase activity (n = 5). **E** Hippocampal neurons were pretreated with 1 μM PLC inhibitor—U73122 or **F** 1 μM sarco(endo)plasmic Ca^2+^-ATPase inhibitor—thapsigargin or **G** 20 μM ryanodine receptor inhibitor—ryanodine (n = 5 for each) and luciferase activity was quantified 12 h following BDNF stimulation. **H** Colocalization of AKAP6 and ryanodine receptor 2 (RyR2) in primary hippocampal neurons. Fixed cells were immunostained with anti-AKAP6 primary and respective Alexa Fluor 594 conjugated secondary antibody (shown in red), as well as with anti-RyR2 primary antibody and respective Alexa Fluor 488 conjugated secondary antibody (shown in green). Colocalization percentage (fraction of AKAP6-positive pixels which are also positive for RyR2) was calculated using Leica LAS AF Lite software. Scale bars: 20 μM. **I** The design of CaN anchoring disruptor. The sequence corresponding to AKAP6 CaN binding domain (aa 1286–1345, CBD) was subcloned into rAAV-CAG-EGFPSV40 vector for AAV2 production. **J** Hippocampal neurons were co-transduced with NFAT dual-reporter lentivirus and AAV2-CBD-GFP (or AAV2-GFP control) for assessment of NFAT transcriptional activity (n = 5). **K** In vitro survival of RGC transduced with AAV2-CBD-GFP or AAV2-GFP alone, or **L** electroporated with NFATc4 or control siRNA and cultured in media with or without BDNF, normalized to appropriate control-treated RGCs cultured in minimal media (no forskolin, no BDNF). Representative data from repeated experiments shown (n = 3). **M** Experimental design of the optic nerve crush (ONC) in vivo model. BDNF was intravitreally injected immediately following ONC, and retinas were collected 7 days after. **N** Quantification of BDNF effect on RGC survival in vivo following ONC in wild-type (WT) or Nfatc4^−/−^ mice. Retinal ganglion cells were stained with anti-RBPMS antibody and counted manually. Data are expressed as the ratio of RBPMS-positive cells in the BDNF-treated eye relative to control (PBS-injected) eye (n = 4 per group). The RGC densities (cells per mm^2^) in the Sham-operated groups are as follows: 3243 ± 89 for WT and 3159 ± 57 for Nfatc4^−/−^ mice. **O** The schematic model of AKAP6-dependent regulation of NFAT transcriptional activity in response to BDNF stimulation. *P < 0.05, **P < 0.01, ***P < 0.001. Error bars indicate standard error of mean (± SEM)

Using primary hippocampal neurons, we demonstrate here that a 20-min stimulation with BDNF increases CaNAα and NFATc4 binding to AKAP6 (Fig. 1A, B), indicating neurotrophin-mediated anchoring of both proteins within the same signaling complex. Since NFAT activation requires CaN-mediated dephosphorylation, we anticipate that scaffolding of CaNAα and NFATc4 by AKAP6 can serve as a nodal point for BDNF-mediated regulation of NFAT transcriptional activity.

To explore this possibility, we knockdown AKAP6 using lentiviral-mediated shRNA delivery (73 ± 4% knockdown efficiency as assessed by real time PCR, not showed) and observed a significant reduction in BDNF-mediated NFAT-dependent transcription (Fig. 1C). Next, we verified that the effect of BNDF on luciferase activity [6] was dependent on NFATc4. Knockdown of NFATc4, with 81 ± 6% efficiency (not showed) attenuated NFAT transcription by 60 ± 9%, highlighting NFATc4’s role in AKAP6-mediated BDNF action (Fig. 1D). Further, we examined signaling pathways upstream to AKAP6/NFATc4 with regard to their role in triggering NFAT-dependent transcription (Fig. 1E–G). Consistent with the canonical mode of BDNF action, pretreatment with phospholipase C (PLC) inhibitor U73122 decreased NFAT transcriptional activity. Similar effect was observed when intracellular Ca^2+^ stores were depleted with thapsigargin or ryanodine receptor (RyR)-mediated Ca^2+^ release was blocked by ryanodine. Furthermore, AKAP6 colocalized with RyR2 in hippocampal cultures (Fig. 1H) suggesting the importance of PLC-RyR signaling in the AKAP6-dependent orchestration of NFATc4 activation in response to BDNF.

To verify whether CaN anchoring to AKAP6 is necessary for BDNF-mediated NFATc4 activation we used a displacing peptide corresponding to CaN binding site in AKAP6 (aa 1286–1345, CBD, Fig. 1I). Transduction of hippocampal neurons with adeno-associated virus (AAV2)-GFP-CBD decreased both basal and BDNF-mediated NFAT activation (Fig. 1J). Because our results indicate that BDNF signals through AKAP6-CaN-NFATc4, we next assessed whether anchoring of CaN and NFATc4 to the scaffold is required for BDNF-mediated neuronal survival. For this purpose, we switched to RGCs, as their culturing and manipulation in vitro produces stress and subsequent ongoing cell death even in neurotrophic factor-rich growth media. No changes in RGC survival were seen in minimal media or in media with forskolin alone. However, the pro-survival effect of BDNF was significantly attenuated in neurons expressing AAV2-GFP-CBD when compared to GFP control (Fig. 1K). Similarly, siRNA-mediated NFATc4 knockdown resulted in significant decrease of BDNF effect (Fig. 1L).

Finally, to conclusively demonstrate that NFATc4 is required for BDNF-mediated neuroprotection in injured neurons, we performed ONC procedure in NFATc4^−/−^ mice and collected retinas a week after BDNF injection (Fig. 1M) [7]. The ONC is an effective preclinical model for neuronal trauma and regeneration failure, as it similarly severs all of the RGC’s axons and induces time-dependent retrograde RGC death [8–10]. Staining of flat mount retinas with RBPMS demonstrated that survival of BDNF-treated RGCs, expressed as BDNF/control (PBS) ratio was increased 61 ± 9% in wild-type mice (Fig. 1N). However, no prosurvival effect of BDNF intravitreal injection was observed in NFATc4^−/−^ mice after ONC.

## Discussion and future perspective

Neurotrophic factors play a crucial role in promoting the survival of neurons by activating various signaling pathways, some of which overlap while others are distinct [11]. However, the spatial organization of these pathways, particularly in ensuring neurotrophin-mediated survival, has remained unclear. As summarized in Fig. 1O, AKAP6 may provide a suitable platform for anchoring CaN and NFATc4 in the perinuclear compartment, thus allowing for spatial control of NFATc4 which is downstream to BDNF-RyR-Ca^2+^-dependent neuroprotection. This provides a novel understanding of the mechanisms behind BDNF's pro-survival effects. Because BDNF also acts through ERK5 [12], ERK5 is anchored to AKAP6 through PDE4D3 [13] and it phosphorylates NFATc4 on S(168,170) [14], we cannot exclude the involvement of ERK5 in the regulation of BDNF-dependent NFAT activity. While our previous findings did not explore the effect of BDNF, evidence from peptides delocalizing signalosome constituents strongly indicates AKAP6 as potential transducer of neurotrophic signaling crucial for RGC survival post-trauma or insult [4]. Considering AKAP6 as a platform for pro-survival signal crosstalk, it cannot be ruled out that PKA and CaN/NFATc4 signaling at the signalosome may synergistically counterbalance retrograde axonal death following injury. However, understanding the balance of these pathways within the AKAP6 complex for different neuronal functions (e.g., survival vs. outgrowth) under physiological conditions or following injury remains a significant gap, which deserves further studies. Such investigations hold promise for valuable insights that could contribute to the development of neuroprotective strategies, aiming to preserve injured neurons from degeneration and facilitate regeneration.

## Supplementary Information

Supplementary Material 1

## Data Availability

All data and materials are available from the corresponding author upon reasonable request.

## Abbreviations

BDNF: Brain-derived neurotrophic factor
AKAP: A-kinase anchoring protein
RGC: Retinal ganglion cell
CaN: Calcineurin
NFAT: Nuclear factor of activated T cells
MAPK: Mitogen-activated protein kinase
PKA: Protein kinase A
PLC: Phospholipase C
RyR: Ryanodine receptor
AAV: Adeno-associated virus
ONC: Optic nerve crush
RBPMS: RNA-binding protein with multiple splicing
PDE4D3: Phosphodiesterase 4D3
ONC: Optic nerve crush
RBPMS: RNA-binding protein with multiple splicing
