# Supplementary material for: AKAP6 controls NFATc4 activity for BDNF-mediated neuroprotection
Source: Mol Brain. 2024 Nov 22;17:85. doi: 10.1186/s13041-024-01157-8 (PMC11585144; doi:10.1186/s13041-024-01157-8)
Supplement: Supplementary file 1 — Supplementary Material 1 [file 13041_2024_1157_MOESM1_ESM.docx]

**Supplementary File 1- Detailed materials and methods**

**Animals**

All animal procedures were carried out according to the Association for Research in Vision and Ophthalmology (ARVO) guidelines for the use of animals in ophthalmic and vision research. The experimental protocols were approved by the Institutional Animal Care and Use Committee at the Medical University of Lodz. Primary hippocampal neurons and retinal ganglion cells were isolated from Sprague-Dawley rats as described in the following sections. The animals were group-housed in laboratory cages and kept under a controlled temperature (23 ± 2 °C) with a 12-h light/dark cycle and with food and water provided ad libitum. Nfatc4^-/-^ mice (B6;129S-*Nfatc4*^tm1Grc^/J, strain #027581) were from the Jackson Laboratory (USA). Both males and females were used for in vivo experiments.

**Reagents**

If not specified otherwise, reagents were purchased in Thermo Fisher (USA). Xylazine and ketamine were purchased from BioWet (Poland), annexin V-FITC staining kit was from Abcam (UK), ECL substrates was from Bio-Rad (USA), Protein A/G PLUS-Agarose was from Calbiochem (USA), BDNF was from Abnova (Taiwan), Insulin and poly-D-lysine were from Merck (USA).

Lentiviruses were produced by Genewiz from Azenta Life Sciences (Germany). The shRNA sequence used for rat AKAP6 knockdown (NCBI GI:5070430, base pairs 7210–7228) was as follows: AKAP6 shRNA (sense strand), 5′-GACGAACCTTCCTTCCGAATTCAAGAGATTCGGAAGGAAGGTTCGTCTTTTT-3′, as well as control shRNA (sense strand), 5′-GACGAACCCCTGTTCCGAATTCAAGAGATTCGGAACA GGGG TTCGTCTTTTT-3′, which was previously published [4] and shown by us to efficiently diminish cAMP synthesis at the AKAP6 compartment [4]. Target and control shRNA, driven by the U6 promoter, were cloned into the LentiCRISPRv2-mCherry lentiviral vector (Addgene Plasmid #99154). This vector also contained mCherry driven by the EF-1α promoter to control for shRNA expression. Rat shRNA (4 unique 29mer constructs) for Nfatc4 knockdown (NM_001107264, NM_001107264.1), cloned into the pGFP-C-shLenti plasmid, was commercially available from Origine (USA). The Lenti-X packaging system was used to produce lentiviral particles with a titer ranging from 10^7 to 10^8 TUs/ml. Adeno-associated viruses serotype 2 (AAV2) carrying the AKAP6 fragment (aa1286-1345) corresponding to the calcineurin-binding domain (CBD) were prepared by BioHippo Inc. (USA). The sequence for overexpression of the CBD of AKAP6 (referred to here as GFP-CBD) was initially cloned into pmCherry-1286-1345 and subsequently subcloned into the rAAV-CAG-EGFPSV40 vector for AAV2 assembly and purification. The titer was 2.06 × 10^12 viral/ml.

**Isolation of primary hippocampal neurons and retinal ganglion cells**

Retinal ganglion cells (RGCs) were isolated from postnatal (P2-P4) Sprague-Dawley rats using the method described in our previous paper (4). In brief, isolated retinas were washed three times with PBS and digested with papain (16.5 U/ml) for 30 min at 37°C. Next, papain activity was inhibited with ovomucoid (1.5 mg/ml), and the cell suspension was centrifuged at 250 x g for 10 min. The pellet was suspended in Dulbecco’s-PBS supplemented with 5 μg/ml insulin and transferred to anti-macrophage antibody-coated petri dishes for a 45-min incubation. Cells were subsequently transferred to petri dishes containing anti-Thy 1.2 antibody-conditioned media and further incubated for 45 min to isolate RGCs. Following purification, RGCs were seeded at a density of 1000-2500 cells/well in a 24-well plate coated with poly-D-lysine (10 μg/ml) and laminin (1 μg/ml). The cells were cultured in Neurobasal serum-free media supplemented with sodium pyruvate (1 mM), B27 (1:50), BDNF (50 ng/ml), CNTF (10 ng/ml), forskolin (5 mM), insulin (5 μg/ml), N-acetyl cysteine (5 μg/ml), L-glutamine (1 mM), and triiodothyronine (40 ng/ml), unless otherwise specified, at 37°C/5% CO_2_ in a humidified atmosphere. The purity of the isolated RGCs was checked with anti-RBPMS antibodies and typically exceeded 95%.

Primary hippocampal neurons were isolated from E18 Sprague-Dawley rat embryos as described previously (4). In brief, hippocampi (CA1-CA3) were dissected on ice in HBSS buffer, trypsinized for 30 min at 37^0^C, centrifuged at 250 x g for 2 min, and triturated with a fire-polished glass pipette. The dissociated neurons were seeded on glass coverslips coated with poly-L-lysine in plating medium (10% v/v horse serum in DMEM). Four hours after plating, the medium was exchanged with maintenance medium (Neurobasal supplemented with 2% B27, 1 mM glutamine, 1 mM sodium pyruvate, and 5 μg/ml insulin). The purity of culture was assessed by co-staining with mouse monoclonal anti-MAP2 (neuronal marker, 1:1000, Catalog # 13-1500, Thermo Fisher) and rabbit polyclonal anti-GFAP (glial marker, 1:1000, Catalog # AFP-001, Alomone Labs) antibodies.

**Luciferase activity analysis**

NFAT transcriptional activity was assessed following a protocol similar to (13) with some modifications. Briefly, lentiviral particles were engineered to carry a firefly luciferase gene controlled by the NFAT response element positioned upstream of the minimal TATA promoter. Neurons were transduced with Lenti-NFAT luciferase reporter and Lenti-luciferase at DIV0, followed by culturing the neurons for 3 days. NFAT transcriptional activity was assessed in cell lysates using the Dual-Glo Luciferase Assay System (Promega), following the manufacturer's protocol. The expression of the NFAT luciferase reporter was standardized to the expression of firefly luciferase. The fold increase of standardized NFAT luciferase reporter expression was then calculated relative to baseline values. Expression of the firefly luciferase reporter was normalized to the expression of Renilla Luciferase, used as an internal control. To evaluate the impact of AKAP6 or NFATc4 knockdown on NFAT luciferase activity, neurons were typically transduced with lentiviruses carrying shRNA for AKAP6 or NFATc4 at an MOI of 5-10 on DIV3-DIV4. For intracellular signaling inhibitors, hippocampal neurons were pre-treated with 1 μM thapsigargin, 1 μM U73122 or 20 μM ryanodine for 30 min before BDNF stimulation. Luciferase activity was assayed 12 h later.

**Co-immunoprecipitation**

For co-immunoprecipitation assay, hippocampal neurons were treated with BDNF (100 ng/ml) for 20 min and then 200 μg of proteins was pre-cleared with 20 μl of Protein A/G PLUS-Agarose beads for 2 h at 4°C and centrifuged at 9400 rpm for 5 min. The pre-cleared supernatant was incubated with polyclonal goat anti-AKAP6 antibodies (~2 μg of antibodies/200 μg of lysate proteins, Catalog # NB300-869, ABclonal) overnight at 4°C followed by an incubation with 25 μl of Protein A/G PLUS-Agarose beads at 4°C for 2h. Normal mouse IgG (Catalog # AC011, ABclonal) at the same concentration was used as a negative control. The immunocomplexes were recovered by centrifugation at 9400 rpm for 5 min, washed three times with PBS, eluted with 60 μl of SDS-PAGE sample buffer (62.5 mM Tris-HCl, pH 6.8, 10% glycerol, 2% SDS, and 0.001% bromophenol blue) containing 5% β-mercaptoethanol and subjected to immunoblotting. The membranes with immunoprecipitated AKAP6 protein were probed with polyclonal rabbit anti-CaNAα (1:500, Catalog # sc-9070, Santa Cruz Biotechnology) or polyclonal rabbit anti-NFATc4 (1:500, Catalog # AV32715, Merck) antibodies followed by goat anti-rabbit secondary antibodies (1:20 000, Catalog # AS014, ABclonal) conjugated to horseradish peroxidase. Bands were visualized using ECL system according to the manual provided by the manufacturer. The results were normalized to OD/mg protein and are presented as the fold change in relation to vehicle-treated cells.

**Immunocytochemistry**

Hippocampal neurons were fixed using 4% paraformaldehyde in ice-cold PBS for 30 minutes. Following several washes with PBS, cells were permeabilized with 0.05% Triton X-100 for 15 minutes at 4°C and blocked with 6% BSA for 2 hours at room temperature. The coverslips were then incubated overnight with primary antibodies at 4°C. The following antibodies were used: polyclonal goat anti-AKAP6 (diluted 1:150 in 1% BSA in PBS, Catalog # NB300-869, ABclonal) and polyclonal rabbit anti-RyR2 (diluted 1:50 in 1% BSA in PBS, Catalog # ARR-002, Thermo Fisher). Cells were subsequently probed for 3 hours at room temperature with secondary antibodies conjugated with Alexa Fluor 594 donkey anti-goat IgG (for AKAP6, diluted 1:1000 in 1% BSA in PBS, Catalog # A-11058, Thermo Fisher) or Alexa Fluor 488 goat anti-rabbit (for RyR2, diluted 1:1000 in 1% BSA in PBS, Catalog # A-11008, Thermo Fisher). The coverslips were mounted using Vectashield mounting medium. Images were captured on a TCS SP5 confocal laser-scanning microscope equipped with a 63× objective (Leica). The degree of colocalization was assessed with Leica LAS AF Lite software. None of the immunofluorescence reactions revealed unspecific fluorescent signals in the negative controls.

**In vitro RGC survival**

Isolated RGCs (~150,000 cells/well of a 6-well plate) were immediately electroporated with NFATc4 ON-TARGETplus siRNA oligonucleotides or ON-TARGETplus scrambled siRNA, both administered at 1 nmol per electroporation (Horizon Discovery). The electroporation was conducted according to the method detailed elsewhere [12]. Another portion of RGCs was transduced 3 h after seeding with AAV2-CBD-GFP or AAV2-GFP at 1000 MOI, followed by a 1:1 media change the next day. To investigate the role of NFATc4 and AKAP6-CaN anchoring in BDNF signaling, we cultured RGCs in growth media containing either forskolin (5 μM) alone or in combination with BDNF (100 ng/ml) for up to 3 days. Subsequently, cells were double-stained with annexin V-FITC for 10 min in growth media to label apoptotic cells and Calcein Red™ for 20 min to label live cells. Images of 10-12 randomly selected fields were captured in growth media using a Leica DMi8 inverted microscope to quantify cell survival. Survival was quantified using ImageJ. All Calcein-positive, annexin V-negative neurite-extending cells were scored as live, and all Calcein-negative, annexin V-positive cells were scored as dead RGCs.

**Optic nerve crush and RGC survival in vivo**

Optic nerve crush, retina flat mount, and RGC quantification were performed essentially as outlined in a previously published protocol (14). Briefly, mice were anesthetized with a combination of 20 mg/kg xylazine and 100 mg/kg ketamine administered intraperitoneally (IP). Optic nerve crush involved exposing the left optic nerve from the outer canthus and gently compressing it for 5 s, approximately 1.5 mm behind the eye globe (3). Care was taken to avoid damage to the retinal blood supply. Immediately after optic nerve crush, distinct groups of mice were administered either BDNF (5 μg) dissolved in 3 μL of PBS, while control mice received PBS alone. Mice exhibiting significant postoperative complications such as retinal ischemia or cataracts were excluded from further analysis. The eyes were removed and fixed with 4% PFA for 2 h at room temperature. Retinas were dissected and permeabilized for 1 h in flat mount blocking buffer (3% Triton X-100, 0.5% Tween-20, 1% BSA, 4% sodium azide in PBS). Subsequently, retinas were incubated at 4°C for 48 h with a rabbit polyclonal anti-RBPMS antibody (1:250 in flat mount blocking buffer, Catalog # PA5-116434, Thermo Fisher). After three washes with PBS, retinas were incubated overnight with Abflo® 594-conjugated goat anti-rabbit IgG (1:500 in flat mount blocking buffer, Catalog # AS039, ABclonal). Nuclei were stained with DAPI (1:5000) in PBS for 15 min. Flat-mounted retinas were then mounted in an antifade mounting medium (EverBrite™ Hardset Mounting Medium, Biotium). Images were acquired using a Leica DMi8 inverted microscope with a x10 magnification objective. For quantification, retinas were divided into four quadrants at a fixed distance from the optic nerve head, and one image was captured from each area. RBPMS-positive cells were manually counted in a masked fashion.

**Statistics**

Statistical analysis was done in GraphPad Prism. To compare two samples two-tailed Students t-test was conducted, for more than two sample comparisons ANOVA with Tukey’s post-hoc analysis was done. * P<0.05, ** P<0.01, *** P<0.001.
